# Evolution of Hsp70 Gene Expression: A Role for Changes in AT-Richness within Promoters

**DOI:** 10.1371/journal.pone.0020308

**Published:** 2011-05-31

**Authors:** Bing Chen, Tieliu Jia, Ronghui Ma, Bo Zhang, Le Kang

**Affiliations:** Institute of Zoology, Chinese Academy of Sciences, Beijing, China; California State University Fullerton, United States of America

## Abstract

In disparate organisms adaptation to thermal stress has been linked to changes in the expression of genes encoding heat-shock proteins (Hsp). The underlying genetics, however, remain elusive. We show here that two AT-rich sequence elements in the promoter region of the *hsp70* gene of the fly *Liriomyza sativae* that are absent in the congeneric species, *Liriomyza huidobrensis*, have marked *cis*-regulatory consequences. We studied the *cis*-regulatory consequences of these elements (called *ATRS1* and *ATRS2)* by measuring the constitutive and heat-shock-induced luciferase luminescence that they drive in cells transfected with constructs carrying them modified, deleted, or intact, in the *hsp70* promoter fused to the luciferase gene. The elements affected expression level markedly and in different ways: Deleting *ATRS1* augmented both the constitutive and the heat-shock-induced luminescence, suggesting that this element represses transcription. Interestingly, replacing the element with random sequences of the same length and A+T content delivered the wild-type luminescence pattern, proving that the element's high A+T content is crucial for its effects. Deleting *ATRS2* decreased luminescence dramatically and almost abolished heat-shock inducibility and so did replacing the element with random sequences matching the element's length and A+T content, suggesting that *ATRS2*'s effects on transcription and heat-shock inducibility involve a common mechanism requiring at least in part the element's specific primary structure. Finally, constitutive and heat-shock luminescence were reduced strongly when two putative binding sites for the *Zeste* transcription factor identified within *ATRS2* were altered through site-directed mutagenesis, and the heat-shock-induced luminescence increased when *Zeste* was over-expressed, indicating that *Zeste* participates in the effects mapped to *ATRS2* at least in part. AT-rich sequences are common in promoters and our results suggest that they should play important roles in regulatory evolution since they can affect expression markedly and constrain promoter DNA in at least two different ways.

## Introduction

Phenotypic differences between species can be due to genetic changes that alter gene products as well as their expression level [1], [2], [3], [4]. Many workers have identified regulatory mutations that alter existing regulatory elements or create new ones, often with significant consequences for morphology, physiology, and behavior that are mediated by quantitative and spatio-temporal changes in gene expression [2], [3], [5], [6]. Understanding the genetic and molecular mechanisms involved in regulatory divergence is therefore expected to provide important insights into phenotypic evolution.

Identifying the cis-regulatory changes underlying expression differences between species, however, remains challenging both experimentally and bioinformatically [4], [6], [7]. This is also true for the AT-rich sequences frequently found in eukaryotic promoter regions [8], [9], [10] that vary substantially across taxa with respect to abundance and organization. Indeed, no essential sequence motifs have been characterized in such regions that are shared by many taxa and whose presence was linked with regulatory consequences for gene expression [11], [12], [13].

In many species, large-scale analysis of GC-content of individual genes revealed sharp peaks in A+T content near transcribed DNA [11], with the promoter regions of genes being AT-richer than their coding regions and containing long and short AT-rich stretches [9], [14]. Many studies show that the AT-rich DNA in promoter regions should affect the regulation of chromatin, transcription-factor binding, and gene expression [13], [15], [16], [17]. For example, AT-poor and AT-rich chromosomal regions have different patterns of chromatin compaction and of histone modification. Some AT-islands function as Matrix Attachment Regions (MARs) whose association with the nuclear matrix may define the borders of chromatin domains and mediate the regulation of transcription [14]. A few AT-rich sequence elements in promoter regions have been found to contain transcription-factor binding sites with demonstrable regulatory impact [16], [18], [19], [20]. Furthermore, comparative-genomics evidence shows that the organization of AT-rich sequences of orthologous genes diverges during evolution [11]. However, to the best of our knowledge there is no published evidence that the varying composition and organization of AT-rich sequences in promoter regions correlate with gene expression differences between species or higher taxa.

The expression of heat-shock protein (Hsp) genes is an established model phenotype for the study of the evolutionary significance of regulatory mutations in response to environmental change [1], [2], [3], [5], [21]. Hsps are molecular chaperones that help client proteins to recover proper folding when protein folding is perturbed by heat and other stress factors and help to initiate the degradation of misfolded proteins [22], [23]. Hsps have been shown to increase markedly the resistance to thermal and oxidative stress in *D. melanogaster* and other species [22], [23], [24]. Distinctive features of the transcriptional machinery involved in Hsps expression facilitate the rapid and massive expression of Hsps in response to thermal stress [1], [25]. Nevertheless Hsp genes show distinct evolution of their expression pattern. Variation in *hsp* expression in natural populations seems to correlate at least partially with changes in *hsp* promoters. For example, *hsp* promoter regions are highly susceptible to transposable-element insertions which often have large effects on expression [1], [5]. The regulatory regions of *hsp70* and of other *hsp*s in some natural populations of *D. melanogaster* show clear divergence caused by such insertion/deletions, and this divergence is correlated with differences in *hsp* expression [1], [5], [7], [26]. The expression changes of *hsp* have been shown to affect individual fitness [5], [25], [27], to be linked to adaptive response to thermal stress, and to contribute to incipient speciation in certain environments [21], [26].

Through comparative study of two congeneric species, *Liriomyza huidobrenssis* and *Liriomyza sativae*, we have found previously a link between the expression pattern of *hsp* and the evolutionary divergence of the response to thermal stress[21]. The coding sequences of the orthologous *hsp*s of these two species are very similar, e.g., the amino acid sequences encoded by their *hsp70*s are 97% identical, but between the two species we found marked differences in how these genes' expression responds to a range of low and high temperature exposures [28]. Moreover, the temperature for onset and maximal induction of *hsp* expression of either species was consistent with the extreme thermal environments experienced by them [24], [28]. These results suggest that the pattern of *hsp* expression of the two *Liriomyza* species contributes to the species' diverged thermal stress tolerance [21], [28]. The above findings indicate that *hsp*s in these two related species are a favorable system to investigate the genetics of regulatory evolution.

Here we report that the promoter regions of the two *hsp70* orthologs differ in their AT-rich sequences and we study whether these differences correlate with gene expression differences. Indeed, we identified two AT-rich sequence stretches in the *hsp70* promoter that are present in *L. sativae* and may explain much of the differences in *hsp* expression level and heat inducibility observed between this species and *L. huidobrensis*. We characterized indeed the effects of these elements on transcription and zoomed in on some primary-structural features of the elements that may be crucial for their regulatory impact *in vivo*. Because AT-rich sequences account for a large proportion of the promoter region of these *hsp*s, the results suggest that changes in the AT-rich sequences of promoter regions may contribute often to the differentiation of gene expression level and inducibility in closely related species, so that such changes may participate frequently in the evolution of gene expression.

## Results

### Characterization of promoter region of hsp70 orthologs in two Liriomyza species

The intergenic genomic region at the 5′ end of the *hsp70*s was determined in the two *Liriomyza* species through genomic DNA walking. This allowed us to isolate for sequence analysis ∼920 bases of the upstream non-coding region of *Lhuhsp70* and ∼1170 bases of the same region of *Lsahsp70*, both of which included the 5′UTR and the promoter. To characterize the promoters, we first contrasted their A+T content with that of the first 200 bases of the coding region of the two species using a 100-bp sliding window (Figure S1). The two non-coding regions were found to be very AT-rich, averaging 73.5% A+T in *Lhuhsp70* and 73.0% in *Lsahsp70*. The 5′UTRs of the two h*sp70* are 72% similar in sequence but the promoter regions show a lower 68% sequence identity and are AT-richer in *Lsahsp70* than in *Lhuhsp70* (whereas the coding regions' A+T is 56% and 59%, respectively and they are 86% identical).

**Figure 1:**
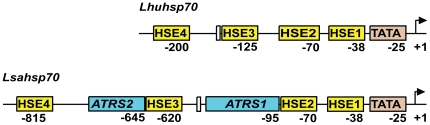
Schematic structure of the promoter region of *Lhuhsp70* and *Lsahsp70* gene. *Lhuhsp70* and *Lsahsp70* are the *hsp70* orthologs of *L. sativa* and *L. huibrobensis*. Two AT-rich sequence elements *ATRS1* (495 bp) and *ATRS2* (98 bp) are present only in *Lsahsp70*. The arrow indicates the transcription start site. The position numbers flag each element's first most downstream base relative to the transcription start site. “HSEn”: Heat-shock element (n = 1–4); “TATA”: TATA box; “*ATRS1* and *ATRS2”*: AT-rich elements; The unlabeled box: GAGA element.

The *hsp70* transcription start site (initiator) was determined by comparing sequences of the promoter region and the full-length cDNA transcript (GenBank accession number AY842476.2 for *L. huidobrensis* and AY842477 for *L. sativae*; [27]). Both TATA boxes are located 30 bp upstream from the initiator sequence. Heat-shock response elements (HSE) are conserved sequences in the promoter regions of *Hsp*s and their binding by the Heat-shock factor (HSF) is central to the heat-shock-induced activation of the genes [29], [30]. We identified four putative HSEs in the *hsp70* promoters by looking for the conserved dimer of the 10-bp NTTCNNGAAN sequence characteristic of *Drosophila* HSEs [31]. The sequences of these HSEs are listed in Table S1. The two HSEs at the proximal promoter, HSE1 and HSE2, have conserved sequence composition and position relative to the transcription start site, whereas the two distal ones, HSE3 and HSE4, occupy very different positions in the two species, being dispersed more further upstream in *Lsahsp70* than in *Lhuhsp70* (Figure 1).

Finally and importantly, we observed another difference between the two gene promoters: The *Lsahsp70* promoter contains two AT-rich sequences elements that are absent from *Lhuhsp70*. One of them is 495 bp long and 75% AT-rich and is located between HSE2 and HSE3 and hereafter will be called the “AT-rich sequence 1” (*ATRS1*); and the other is 98 bp long and 65% AT-rich, and is located between HSE3 and HSE4, and hereafter will be named *ATRS2* (Figure 1).

### Orchestration of ATRS1 and ATRS2 in transcriptional regulation

**Figure 2:**
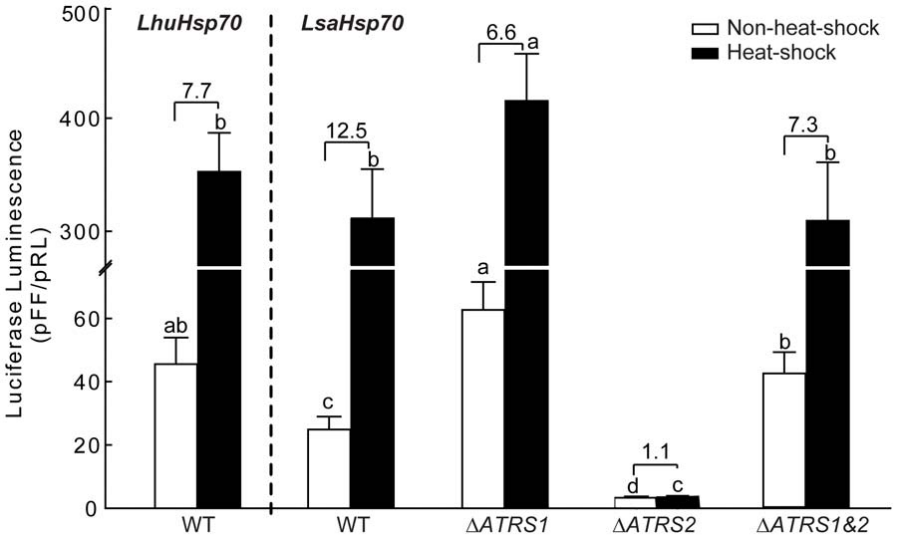
Transient luciferase luminescence driven by constructs carrying an *hsp70* promoter fused to the luciferase gene. The constructs were transfected and expressed in *L. sativae* primary cells with and without heat-shock treatment. Transfected cells were incubated at 25° for 12 h, placed at either 37° (“heat shock”) or 25° (“non-heat-shock”) for 60 min, transferred to a 25° cell incubator for 60 min, and then used for luminescence assays. Luminescence values are the ratio of firefly to *Renilla* luminescence. Constructs with wild-type promoters of the *LhuHsp70* or *LsaHsp70* gene are labeled “WT”; “Δ*ATRS1*” labels a construct with a *LsaHsp70* promoter lacking the *ATRS1* element (see Figure 1); “Δ*ATRS2*” labels one without *ATRS2*; “Δ*ATRS1*&*2*” labels constructs with neither *ATRS1* nor *ATRS2*. The number above each two-column group represents induction fold of luminescence under heat-shock over non-heat-shock condition. The average luminescence over five independent experiments is plotted (mean± one standard deviation (SD)). Different letters above the error bar indicate a significant difference at the 0.05% level within treatments (One-way ANOVA and Turkey's post-hoc test).

We first examined the transcriptional activity of the wild-type promoter of *Lhuhsp70* and *Lsahsp70* using the luciferase assay. In *L. sativae* primary-culture cells, both native promoters showed clear constitutive transcriptional activity, i.e., are luminescent at 25°C, albeit the *Lsahsp70* promoter delivered less luminescence than the *Lhuhsp70* promoter (*P*<0.05; Figure 2). Next, to determine if the two AT-rich insertions affect transcription, we measured the luminescence delivered by constructs carrying a wild-type promoter of *Lsahsp70* out of which both *ATRS1* and *ATRS2* had been deleted experimentally. The double deletion increased luminescence (*P*<0.05), restoring it to that delivered by the wild-type promoter of *Lhuhsp70* (Figure 2). The deletion of *ATRS1* alone resulted in elevated luminescence (*P*<0.05), suggesting that *ATRS1* down-regulates transcription. In contrast, deleting *ATRS2* decreased luminescence sevenfold (P<0.01) relative to that of the native *Lsahsp70* promoter. These results suggest that *ATRS2* acts to up-regulate transcription at the distal promoter (Figure 2).

To examine the regulatory role of *ATRS1* and *ATRS2* in heat-shock response, we measured luciferase luminescence after 37°C heat-shocks. Heat-shock raised the luminescence delivered by the two native promoters up to ten times that of non-heat-shocked cells (Figure 2). Nonetheless, the two native promoters did not deliver significantly different luminescence in the *L. sativae* primary-culture cells at the 37°C treatment. When *ATRS1* was deleted, heat-shock increased the induced luminescence significantly above that of non-heat-shocked cells, whereas deleting *ATRS2* almost abolished heat inducibility, suggesting that *ATRS2* is required to induce *hsp70* expression by heat-shock *in vivo*. The different luminescence levels driven by native and deletion variants of *Lsahsp70* promoters after heat-shock are significantly different (*P*<0.05), which is consistent with what was observed with non-heat-shocked *L. sativae* cells (Figure 2). However, the double mutant's high level of luminescence was close to that of the wild-type, indicating that the effects of two elements are counteractive under heat-shock response. The luciferase assays with another four insect cells S2, Sf9, SpexII-A, and HZ-AML-2 cell lines gave similar results (Figure S2).

### Effects on transcriptional activity of the A+T content of ATRS1 and ATRS2

**Figure 3:**
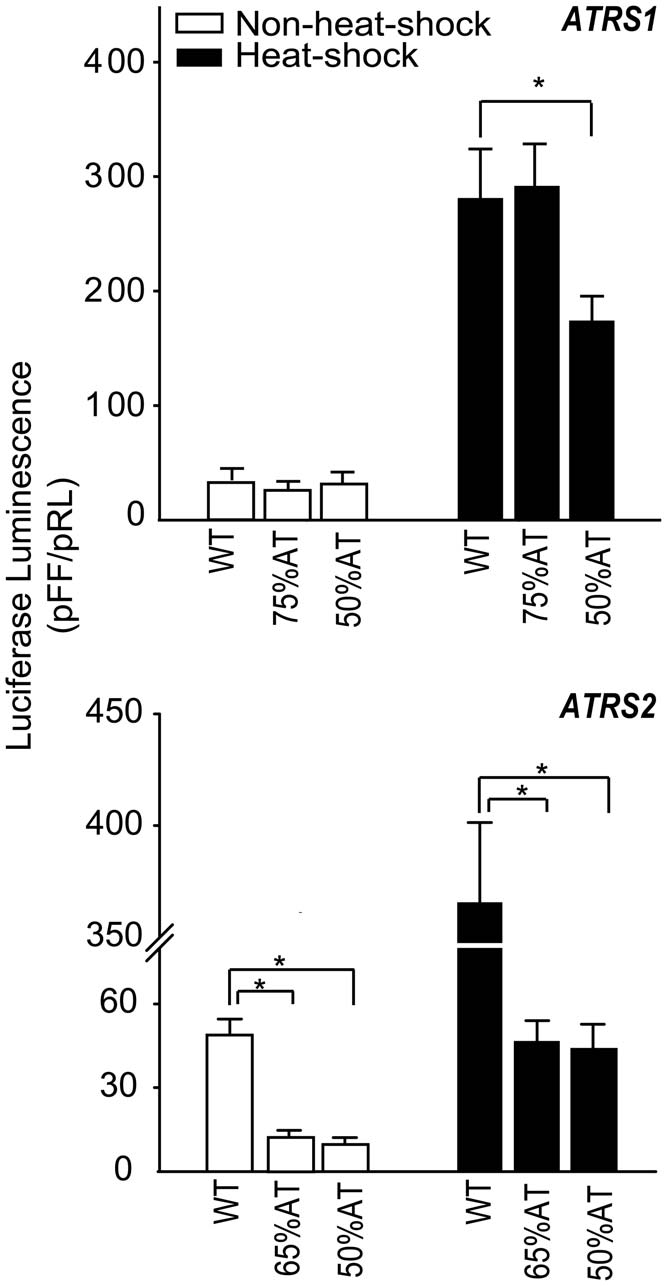
Transient luciferase luminescence driven by constructs carrying *hsp70* promoter with altered A+T content. *ATRS1* or *ATRS2* were replaced with random sequences of the same size but different A+T content; “WT”: construct with wild-type promoter of *Lsahsp70* gene. Methods are as in Figure 2. Luminescence is expressed as in Figure 2. Bars indicate ± one SD. Asterisk (*) indicates a difference significant at the 0.05 level.

To investigate whether the A+T content of *ATRS1* and *ATRS2* contributes to their regulatory activity, we replaced *ATRS1* or *ATRS2* in *Lsahsp70* with random sequences of the same length and various A+T contents, and then assessed the effects on gene expression. Under non-heat-shock conditions, no difference in luciferase luminescence was observed when replacing *ATRS1* with sequences having 50% or 75% A+T content but after heat shock the luminescence was significantly reduced with the 50% AT sequence (*P*<0.05). In contrast and remarkably, swapping *ATRS1* with a sequence of identical A+T content delivered the wild-type transcriptional level and heat inducibility (Figure 3). Therefore *ATRS1* sequence replacements with near-native A+T content can replace the *ATRS1* element at least as far as heat-shock inducibility in transfected cells is concerned.

Contrary to what was observed above, when *ATRS2* was replaced with equally long sequences having 65% (wild-type) or 50% A+T content, luciferase intensity was reduced very similarly across sequences, both under normal conditions and after heat-shock (Figure 3). The two *ATRS2* replacements delivered ∼fourfold and ∼eightfold lower luminosity in non-heat-shocked cells and heat-shocked cells, respectively, relative to the wild type. And upon heat-shock each replacement construct delivered a similarly weaker boost of transcriptional activity than the native promoter. Therefore, *ATRS2’* primary structure rather than mere A+T content is critical for the basal transcription activity and for the heat-shock induced activation of transcription (Figure 3).

### Binding sites for transcription factor Zeste in ATRS2 confer to transcription upregulation

**Figure 4:**
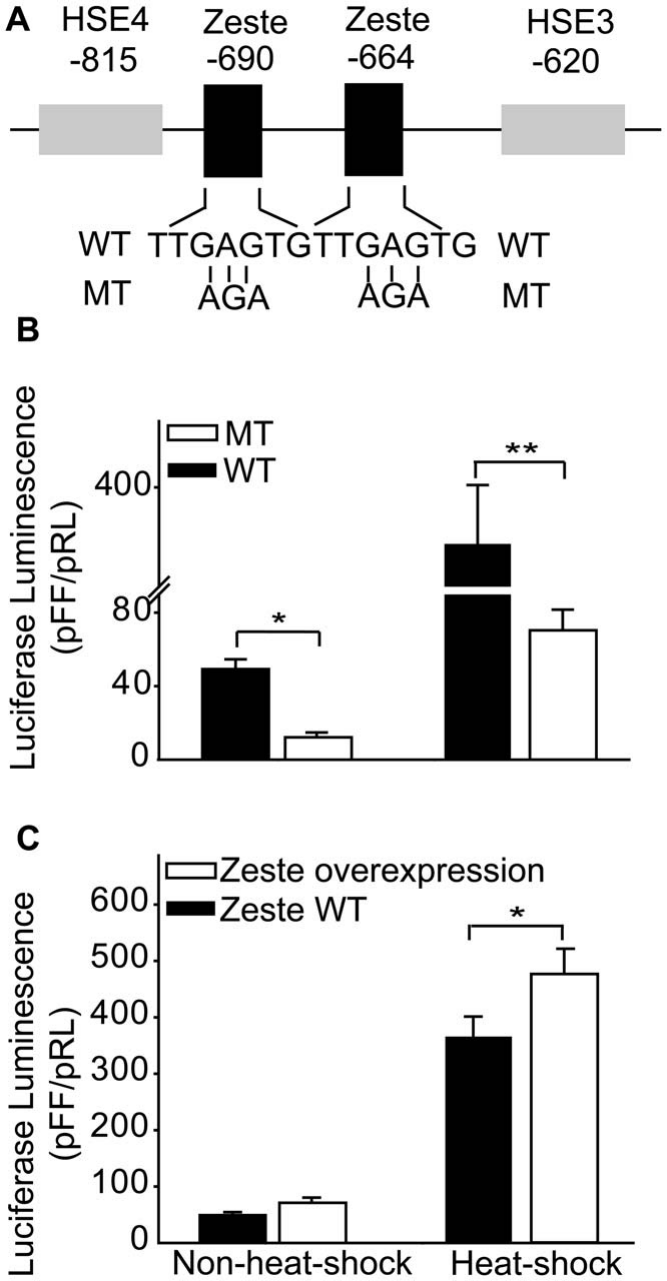
Transient luciferase luminescence and the *Zeste* binding sites predicted within *ATRS2*. (A) Putative binding sites for transcription factor *Zeste* in *ATRS2* of *Lsahsp70*. Mutated sequences (MT) in each putative *Zeste* motif are shown below the wild-type sequences (WT). (B) The effects of mutating the putative *Zeste* binding sites on the luminescence. (C) Luminescence effects of *Zeste* overexpression. To overexpress *Zeste*, expression vectors for *Zeste* or blank control vectors (*Zeste* “wild-type”) were co-transfected together with *Lsahsp70* wild-type reporter plasmids. Bars indicate ± one SD. Asterisks (*) means significance at the 0.05 level and (**) at the 0.01 level.

Homology search in databases for transcription-factor binding sites identified several putative sites for transcription factors in *ATRS2*. Among them were two binding sites for the *Zeste* transcription factor (which is involved in *transvection* in *Drosophila*; [32], [33]). The presence of two such sites in a relatively short region prompted us to study the possible role of these sites in *hsp* regulation. The two putative *Zeste*-binding sites are located between positions -664 and -668 (*Zeste* 1) and between positions -690 and -694 (*Zeste* 2). These sites are relatively GC-rich compared to the flanking DNA (Figure 4A).

To study the functional impact of these sites, we performed site-directed mutagenesis of them, replacing their GAG motifs with AGA ones. The modifications reduced fourfold luciferase luminosity (*P*<0.05) under non-heat-shock conditions and fivefold upon heat-shock (*P*<0.001), relative to that delivered by the native construct (Figure 4B). These two *Zeste* binding sites are therefore essential for transcription activation and heat inducibility driven by the *hsp70* promoter in transfected cells.

To test if *Zeste* is directly involved in the above effects, we co-transfected *L. sativae* primary culture cells with both a vector that expressed the *Zeste* transcription factor and a pGL-3 reporter plasmid containing the wild-type *Lsahsp70* promoter. We observed enhanced luminosity after heat-shock treatment when *Zeste* was overexpressed (*P*<0.05) but no enhancement when control cells were transfected with vectors lacking the *Zeste* gene (Figure 4C).

## Discussion

### Regulatory changes underlie the evolution of hsp70 expression

C*is*- regulatory change, especially change involving transcription-factor binding sites, is viewed as a major source of phenotypic diversity. However, no case of phenotypic evolution has been documented that was due to an alteration of the AT-richness of a regulatory region like a promoter [2], [7]). We hypothesized that change in the base composition and organization of AT-rich sequence elements in promoter regions may be an important contributor to c*is*-regulatory divergence between species, which would be a novel mechanism not requiring the evolution of specific DNA primary structure (unlike the evolution of say a transcription-factor binding site). We studied the ability of the diverged *hsp70* promoter regions of two closely related *Liriomyza* species to drive reporter-gene activity, both at steady state and after heat-shock. Our results indicate that the two AT-rich elements, *ATRS1* and *ATRS2* may contribute pivotally to the *cis*-regulation of diverged pattern of *hsp70* expression between the two species, a pattern which includes repressed steady-state expression and enhanced expression after heat shock. Furthermore, these two regulatory sequences contribute to *L. sativae*'s *hsp70* regulation pattern through different regulation mechanisms as discussed below in detail.

Studying closely related populations or species that show gene expression differences may tell us how adaptation takes advantage of regulatory change[3], [6]. The interspecific differences in transcriptional activity driven by *hsp70* promoters documented here are consistent with the differences in gene expression documented for the two species *in vivo* [28]. For example, *L. sativae* has a similar or lower level of gene expression than *L. huidobrensis* when the flies are subject to stressful temperature ranging from 35°C to 40°C. These two *Liriomyza* species inhabit climate zones posing different thermal challenges [21], [24] and it is likely that they have evolved different adaptations to heat stress, so that tuning Hsp gene expression is likely among these adaptations. Indeed, hsp 70 is a pleiotropic protein that, if expressed say too much, should disrupt cellular homeostasis possibly outweighing the benefits ensuing from better heat shock tolerance [5], [22], [25]. *L. sativae* is abundant in high-temperature zones [24] and its relative low constitutive expression of hsp under normal or even mild heat stress should be favored because too high Hsp expression is detrimental to feeding, development, and reproduction, whereas its higher *hsp70* expression upon heat-shock indicates that it has better adapted to harsh heat stress than *L. huibrensis*, the temperate-climate species [27], [28].

The results reported here, together with our previous studies on *hsp* expression in the same *Liriomyza* species, suggest that changes in the *hsp70* promoter region are very likely to underlie previously documented changes in *hsp70* regulation and that they may contribute in a major way to the two species' divergence in thermal-stress tolerance and its phenotypic correlates. Therefore, divergence in Hsp70 expression regulation is likely to determine the two species' realized thermal niches [3], [21].

### Cis-regulatory effects of AT-rich promoter sequences: repression versus activation

Our analysis of the function of the AT-rich sequence elements of *L. sativae* demonstrates that they can affect transcriptional regulation. Luciferase activity increased when *ATRS1* was deleted, implying that this element may have evolved to inhibit *hsp70* expression. In contrast, deleting *ATRS2* caused a 10- to 100-fold reduction in luciferase activity, indicating that *ATRS2* should enhance constitutive *hsp70* expression. At the same time both elements have different effects on luminescence after heat shock. The promoter with *ATRS1* deletion also drove luciferase expression to a significantly high level. In contrast, deleting *ATRS2* almost abolished the heat-shock response of the promoter in transcriptional activity. Therefore it is likely that evolution of the two elements took place under rigorous selection pressure to keep and perhaps improve heat-shock inducibility.

Experiments with transfected cells from other insects demonstrated that the AT-rich elements have different effects on transcription depending on the cell line in which they are tested and on the thermal regime imposed. These cell lines may differ in their trans-acting factors that can bind to the two AT-rich *cis*-acting elements. Thus, regulatory changes associated with *ATRS1* and *ATRS2* may depend on the trans-acting factors which bind to the two AT-rich sequences and possibly to other motifs in the promoter region (see detailed discussion in following section). Therefore, the two AT-rich sequences *ATRS1* and *ATRS2* play contrasting roles in *hsp70* regulation, and suggest new mechanisms to regulate and evolve Hsp70 expression, e.g., in response to thermal stress. To the best of our knowledge this is the first demonstration that AT-rich sequence elements within a promoter have clear repressive and activating effects in assays with transfected cells, effects that may result in regulatory novelty and regulatory fine-tuning in nature.

### Different mechanisms for transcriptional regulation by the two AT-rich sequences

We used sequence replacements and site-directed mutagenesis to show that the *cis*-regulatory effects of *ATRS1* and *ATRS2* constrain the elements' DNA in different ways. The proximal AT-rich sequence, *ATRS1*, reduced transcription level possibly in the same way as transposable elements do it when they insert themselves in the *hsp70* promoter of *D. melanogaster* [7], [25]. Full-strength transcription of *hsp* requires the orderly interaction of their promoters with the transcriptional machinery as well as proper organization and spacing of the binding sites within the promoters [7], [34], [35]. For example, the spacing between and stereo-alignment of HSEs is critical for transcription as demonstrated by the fact that insertions between HSE1 and HSE2 can reduce promoter activity substantially [35]. Hence, a possible mechanism for the repressive effect of *ATRS1* is that it modifies the promoter region between HSE2 and HSE3 altering promoter architecture and disrupting cooperative binding of heat-shock transcription factors to the *hsp70* promoter [25], [34], [35]. However, *ATRS1*'s high A+T content (75% in average) distinguishes it from most transposable elements and random sequences. The high A+T content appears to be important for the heat-shock inducibility of the promoter activity since after heat shock only high-AT replacement “random” sequences delivered as high an expression level as did the wild-type promoter, while no difference was noticed for constitutive expression (Figure 3). No specific binding sites for known transcription factors were detected with *ATRS1*. Taken together, the above results suggest that *ATRS1* affects transcription through mechanisms that depend to a large extent on *ATRS1*'s high A+T content and its specific length.

In contrast, the deletion of *ATRS2* (the distal AT-rich element) from the *Lsahsp70* promoter reduced dramatically luciferase luminescence. *ATRS2* contains two transcription binding sites for the *Zeste* transcription factor and site-directed mutation of these sites reduced luminescence significantly whereas overexpressing the *Zeste* gene increased luminescence, suggesting that these two *Zeste* binding sites contribute to *ATRS2*'s activation effects. The *Zeste* gene is conserved in *Drosophila* species [33] and there is evidence that its protein is an activator of transcription, e.g., it activates Ubx-promoter constructs in the embryo [36]. Our experiments indicate that *Zeste* sites within *ATRS2* may be required for the proper functioning of the *hsp70* promoter of *L. sativae* and that *Zeste* may up-regulate *hsp70* transcription.

### Evolution of gene expression through AT-rich sequence acquisition in a promoter region

The promoters of the *hsp70s* of the two *Liriomyza* species of interest showed characteristic AT-rich tracts. These promoters are AT-rich in general and show some conserved *cis*-regulatory elements such as HSE but these differ in a major way from the AT-rich tracts. Indeed, *ATRS1* and *ATRS2* are not conserved since they are found only in *L. sativae's hsp70* but not in *L. huidobrensis.* However, AT-rich tracts have been also observed in the promoter region of at least the *hsp70Ba* of *D. melanogaster*, *D. simulans*, and *D. sechellia*, and the specific configuration of these AT-rich tracts within promoters differs across species ([37]; Figure S3). In many species, genome-wide surveys revealed that the AT-content of non-coding DNA near genes is much higher than that of coding DNA [9]. This, together with our results, suggests that differences in the profiles of AT-rich sequences in promoter regions across species may be a major cause of evolutionary divergence in gene expression at least as far as heat-shock genes are concerned.

AT-richness in promoter regions could affect transcription through several molecular mechanisms. One mechanism postulates that AT-rich sequences in promoter region could provide transcription-factor binding sites to transcription factors [16], [18], [19], [20]. We showed here that Zeste binding sites in *ATRS2* are crucial for transcriptional regulation and heat-shock inducibility. AT-richness changes along chromosomes could also affect nucloesome formation, positioning and dynamics [38], [39], [40], as well as chromosome stability [16]. For example, chromatin domains that differ in AT-richness display distinct chromatin conformations and are marked by distinct patterns of histone modifications [15]. The mechanisms by which AT-rich elements like *ATRS1* exert their regulatory effects without need for specific primary structure may involve changes in chromatin and histone structure which in turn affect nucleosome packing and positioning and hence the biophysical accessibility of regulatory DNA for further molecular interactions, but the supporting evidence remains indirect.

Our results demonstrate that AT-rich sequences can influence transcription regulation, and indicate that changes affecting AT-rich sequences in promoter regions, e.g., insertions, deletions, and re-organizations of such elements, may contribute to the evolution of gene expression and thus also to the evolution of higher-level phenotypic differences. As mentioned in the introduction, several features of *hsp* regulation are highly conserved phylogenetically, i.e., are highly intolerant of primary-structural change. The evolution of gene expression to attain short-term adaptive goals may tend to take advantage of changes in the AT-richness of the promoter regions exactly for this reason. AT-rich regulatory DNA sequences indeed vary across species and appear more flexible in sequence composition or length changes than do other regulatory regions controlling Hsp expression [11], [41], and thus they may be more evolvable in transcription regulation [13]. Furthermore AT-rich regulatory elements may facilitate evolutionary fine tuning of gene expression by altering the content and/or the organization of AT-rich sequences in promoter region. We predict that because the promoter regions of most genes in most genomes tend to contain AT-rich sequences, differences in the AT-rich sequences of orthologous genes should be a common observation when comparing closely related species that show diverged expression of the compared genes. It will be interesting to study how such AT-rich sequences evolve and which types of genes tend to rely on such changes when their regulation evolves and which others do it only rarely.

## Materials and Methods

### Cell line preparation and culture

The primary cell culture was isolated from healthy *L. sativae* pupae weighing 20 mg, collected from lab populations. The pupa was swabbed with 70% alcohol and washed three times with Schneider's Insect medium containing high concentration antibiotics (400 IU/ ml penicillin and 400 µg/ml streptomycin). After washing, the sample was minced thoroughly with scissors and kept for 30 min in a 60-mm diameter tissue culture dish with 5 ml of 0.25% trypsin solution (Gibco). The trypsinized tissues were sieved through 100-µm steel mesh to produce a suspension with only single cells. The filtered cell suspension was added to 5 ml of complete Schnerder's Insect medium containing 10% fetal bovine serum (FBS), 200 IU ml/1 penicillin and 200 µg/ml streptomycin, and was then centrifuged at 200×*g* for 10 min. The cell pellet was resuspended in fresh complete Schneider's Insect medium and used to seed 25-cm^2^ tissue-culture flasks that were then incubated at 25°C. After the primary *L. sativae* cell cultures grew to a complete monolayer, cells were washed with 0.02% EDTA-PBS and trypsinized with 0.25% trypsin solution. The subcultures were grown and maintained in fresh Schnerder's Insect medium with 7% FBS, 200 IU/ml penicillin and 200 µg/ml streptomycin.

Other four insect cell lines -S2, Sf9, Spex II-A and HZ-AML-2-- were also subject to transfection and luciferase assays. S2 and Sf9 cells were purchased from Invitrogen, USA. Spex II-A[42] , and HZ-AML-2 cells [43] were kindly provided by Dr. Qilian Qin of the Institute of Zoology, Chinese Academy of Sciences. The Spex-II-A cell line was established starting with fat-body tissue of *Spodoptera exigua* (Lepidoptera: Noctuidae) larvae [43]. Sf9 cells were grown in SFX insect medium and the others in Schnerder's Insect medium, both of which supplemented with 7% fetal calf serum and incubated at 25°C. All the media contained antibiotics (100 IU/mL of penicillin G and 100 µg/mL of streptomycin) and were subcultured every 4 days.

### Genome walking and sequencing of complete hsp70 promoter region

To obtain the DNA template for primary PCR amplification, genomic DNA was extracted from homogenized pupae of the two *Liriomyza* species using the DNeasy Blood & Tissue Kit (Qiagen, Germany) according to the instruction manual. A DNA Walking SpeedUp™ Premix Kit (Seegene, Korea) was used to isolate and characterize the *hsp70* promoter region upstream of the known 5′UTR and the *hsp70* coding region. The walking procedure followed the manufacturer's protocols. Three primers, TSP 1, TSP2 and TSP3 (see Table S2), were designed from the *hsp70* coding sequences of each species [27]. They were paired with DNA-walking ACP primers and with Universal primer provided by the kit for primary, secondary, and tertiary genome-walking amplification. The resulting tertiary PCR products were gel-separated, purified, and sequenced. With the same method, regions further upstream of the promoter region were amplified and sequenced until we characterized the entire 5′ intergenic region between *hsp70* and its upstream unannotated gene (GenBank accession number: GU046393). We confirmed that we had the specific *hsp70* gene copy by test-amplifying with three different primer pairs, with the forward primers targeting different sequences upstream of position -200 and the corresponding reverse primers targeting coding-region sequences. The three amplicons were sequenced and could be aligned successfully to form a continuous sequence. The copies from each species with highest identity of sequence in the coding and the 5′UTR region were considered to be the species' *hsp70* orthologs and were named *Lhuhsp70* and *Lsahsp70* for *L. huidobrensis* and *L. sativae*, respectively. The GenBank accession numbers for the final *hsp70* complete sequences and their promoters are HQ703004 and HQ703003, for *Lsahsp70* and *Lhuhsp70*, respectively.

### Construction of luciferase reporter plasmids

The plasmids with fused promoter and luciferase reporter gene were constructed starting with the promoter/enhancer-free pGL-3 basic vector (Promega). DNA fragments containing various constructs of the *hsp70* promoter region were placed upstream of the luciferase gene in the pGL3-basic vector. The PCR-primer pairs Lhuhsp70F / Lhuhsp70R and Lsahsp70F / Lsahsp70R were used to amplify the wild-type promoters of *Lhuhsp70* and *Lsahsp70* genes, respectively (Table S2). Each amplified promoter begins 65 bp upstream of HSE4 and ends 135 bp downstream of the transcription start site and it encompasses therefore the promoter, the 5′UTR, and the first 55 bp of the *hsp70* coding sequence (Figure 1). Five µg of each PCR product were digested with KpnI and BglII in a large 80 µL reaction, and 4 µg of the pGL-3-Basic Vector (Promega) were digested separately with the same restriction enzymes. Digests were purified with QiaQuick Gel-Extraction kit (Qiagen, Germany). Amplified promoters were ligated into the pGL-3 vector using T4 ligase and then the plasmid was transformed into competent *E. coli* DH5α for amplification. All constructs were confirmed by gel electrophoresis and DNA sequencing.

To remove the AT-rich sequence fragment from *ATRS1* and *ATRS2* (Figure 1) and to create the corresponding “excision” mutants, two *hsp70* promoter fragments were amplified using as template the wild-type *Lsahsp70* promoter in the construct described above. The portion of the *Lsahsp70* promoter upstream from *ATRS1* or *ATRS2* was amplified using the aforementioned Kpn1-site-containing upper primer, Lsahsp70F, and a lower downstream-gene-specific primer containing a Pac1 restriction site (Table S2). The lower primer starts just upstream of *ATRS1* or *ATRS2*. The part of the *Lsahsp70* promoter downstream of the fragment *ATRS1* or *ATRS2* was amplified using the aforementioned BglII-site-containing lower primer, Lsahsp70R, and the gene-specific Pac1-site-containing upper primers, which begin just downstream of the fragment (Table S2). The amplified promoter fragments were digested with Pac1 and the upstream and downstream promoter pieces were ligated to each other at the Pac1 site with T4 Ligase. Successful upstream-downstream ligants were amplified with the aforementioned KpnI- and BglII-site-containing primers and confirmed by gel electrophoresis.

To create ‘‘replacement’’ reporter constructs with different A+T contents, the external DNA sequences that would replace *ATRS1* and *ATRS2* were generated from *D. melanogaster* chromosome in a random way. First a 5 mega–bp long chromosome fragment were randomly picked up from Flybase (www.flybase.org). The candidate DNA sequences with a desired A+T content (±0.3%) and the length of ATRS1 or ATRS2 were then screened based on this fragment, Meanwhile, any region spanning from 300 bp upstream to 200 bp downstream an annotated gene was filtered out, the region that contains possible regulatory and coding sequences of the gene. The landmark data of annotated genes in the chromosome were downloaded from NCBI *D. melanogaster* genome database. Thus the screening outputs only sequences that are located within intergenic regions. Last, we check these sequences in TRANSFAC [44] to make sure that they contain no known or predicted transcription factor binding sites. We then choose randomly among these filtered sequence one as the final control sequence (if there is more than one meeting above requirements). The selected replacement sequences for the following experiments were produced by amplifying intergenic sequences of the same length from *D. melanogaster* chromosome 3R, 3L, and 2R. Four fragments were chosen from position 3R: 8327055–8327550, 3L: 9373081–9373575, 2R: 11265144–11265241, and 3R: 11265576–11265673 (see FlyBase, www.flybase.org). The position numbers above are the limits of the respective intergenic sequences. The four fragments would replace *ATRS1* with ∼75% and ∼50% AT and *ATRS2* with ∼65% and ∼50% A+T content, respectively. Each 3′-complementary primer (Table S2) was designed so that the resulting amplicon would have the same length as the AT-rich region that it would replace. The replacement fragments for *ATRS1* or *ATRS2* were ligated with the element's upstream and downstream fragments as described above, one after the other. The ligands were then digested with KpnI and BglII, ligated into pGL-3 (after digestion with KpnI and BglII), and purified as above. Preparations were confirmed by DNA sequencing.

### PCR based site-directed mutagenesis

PCR-based site-directed mutagenesis was performed to create mutant reporter plasmids in which the two consensus transcription-factor binding motifs in the *ATRS2* of the *Lsahsp70* promoter were altered through substitution mutation with two overlapping primers (Table S2). Each primer carried the 3-bp mutations in one of its binding motifs. High-fidelity enzyme *EasyPfu* polymerase was used when amplifying targets in the vector containing the wild-type *Lsahsp70* promoter construct. The PCR products were digested with enzyme DMT (dimethylterephthalate) for direct-cloning. The same procedure was repeated for amplification and digestion but with the second primer pair introducing the 3-bp mutations in another binding motif and with the template of the new vector coming from previous constructs. For all other details we followed the manual for the Easy Mutagenesis System kit (TransGen, Beijing). The resulting construct were confirmed by sequencing from both directions.

### Transient Transfection and dual Luciferase Assay

Cells at 90% confluence in 96-well plates were transfected with pGL3-basic vector DNA (carrying or not a construct). Both a firefly luciferase reporter gene construct (200 ng) and a pRL-SV40 *Renilla* luciferase construct (10 ng; for normalization) were co-transfected per well. After transfection, cells were incubated at 25°C for 12 h, placed in a cell incubator at either 37°C (heat shock) or 25°C (control) for 60 min, transferred to a 25°C cell incubator for 1 h, and harvested by centrifugation. The luciferase activity was measured using the Dual-Luciferase Reporter Assay System according to the instruction manual (Promega). To examine the effects of *Zeste* binding on *hsp70* transcription, 20 ng of *Zeste* expression vector were co-transfected into cells together with 200 ng of pGL3 reporter plasmid and 10 ng of *Renilla* luciferase plasmid, and then luciferase assays were performed. The *Zeste* expression vector was kindly provided by Dr. Lizhao Chen (Chinese Academy of Medical Sciences, Beijing). Five replicate lines were prepared and assayed for each treatment.

### Sequence data and statistical Analysis

The programs ClustalW [45] and MEGA4.0 [46] were used to align the *hsp70* sequences of *L. sativae*, *L. huidorensis*, and several *Drosophila* species. Potential transcription-factor binding sites in sequences of *ATRS1* and *ATRS2* were predicted using the detection tools available in the TRANSFAC database (http://www.gene-regulation.com; [47]) and the EPD database (http://www.epd.isb-sib.ch/; [48]). Searching was limited to transcription factors associated with RNA polymerase II and restricted to the class *Insecta* or the genus *Drosophila*. Luminescence intensity was analyzed using the SPSS package [49]. One-way ANOVA and Turkey's multiple comparison tests (post-hoc) were used to compare mean values. Independent-samples T tests were used to compare differences between control and heat-shock results. A *P*-value of less than 0.05 was defined as statistical significance.

## Supporting Information

Figure S1 **Luminescence driven by** ***hsp70*** **promoters in cell lines from four insect species.** The four different cell lines are S2, SpexII-A, Sf9, and HZ-AM1-2 (see detailed description in Materials and methods). “WT”: wild-type promoter construct of *LhuHsp70* or *LsaHsp70* gene; “Δ*ATRS1*” labels a construct with a *LsaHsp70* promoter lacking the *ATRS1* element (see Figure 1); “Δ*ATRS2*” labels one without *ATRS2*; “Δ*ATRS1*&*2*” labels constructs with neither *ATRS1* nor *ATRS2*. Values are mean ± one SD. Different letters above error bars indicate 95% significant differences (One-way ANOVA and Turkey's post-hoc test).
(TIF)

Figure S2 **A+T content of the promoter regions of** ***Lhuhsp70*** **and** ***Lsahsp70*** **.** Sliding-window size is 100 bp. “TSS”: the transcription start site; “5′UTR”: 5′ unstranscribed region; “CDS”: coding sequence.
(TIF)

Figure S3 **A+T content of the promoter and coding region of** ***hsp70Ba*** **of three** ***Drosophila*** **species.** Sliding-window size is 40 bp.
(TIF)

Table S1 **Sequences of four HSEs in Lhuhsp70 and Lsahsp70.**
(DOC)

Table S2 **Primer sequences used in Materials and methods.**
(DOC)
