# Supplementary figures and images for: Evolution of Hsp70 Gene Expression: A Role for Changes in AT-Richness within Promoters
Source: PLoS One. 2011 May 31;6(5):e20308. doi: 10.1371/journal.pone.0020308 (PMC3105046; doi:10.1371/journal.pone.0020308)

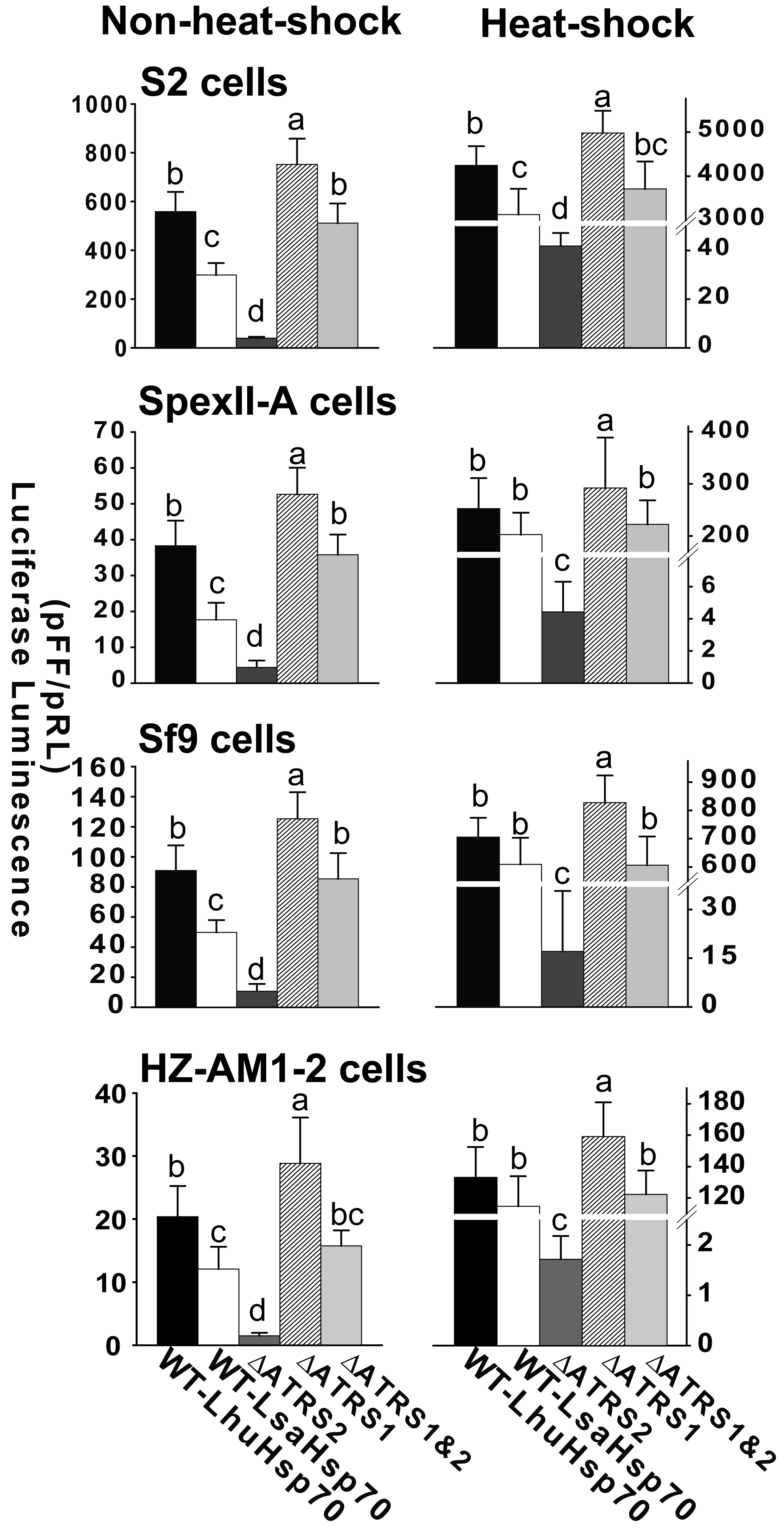

Supplement: Figure S1 — Luminescence driven by hsp70 promoters in cell lines from four insect species. The four different cell lines are S2, SpexII-A, Sf9, and HZ-AM1-2 (see detailed description in Materials and methods). “WT”: wild-type promoter construct of LhuHsp70 or LsaHsp70 gene; “ΔATRS1” labels a construct with a LsaHsp70 promoter lacking the ATRS1 element (see Figure 1); “ΔATRS2” labels one without ATRS2; “ΔATRS1&2” labels constructs with neither ATRS1 nor ATRS2. Values are mean ± one SD. Different letters above error bars indicate 95% significant differences (One-way ANOVA and Turkey's post-hoc test). (TIF) [file pone.0020308.s001.tif]

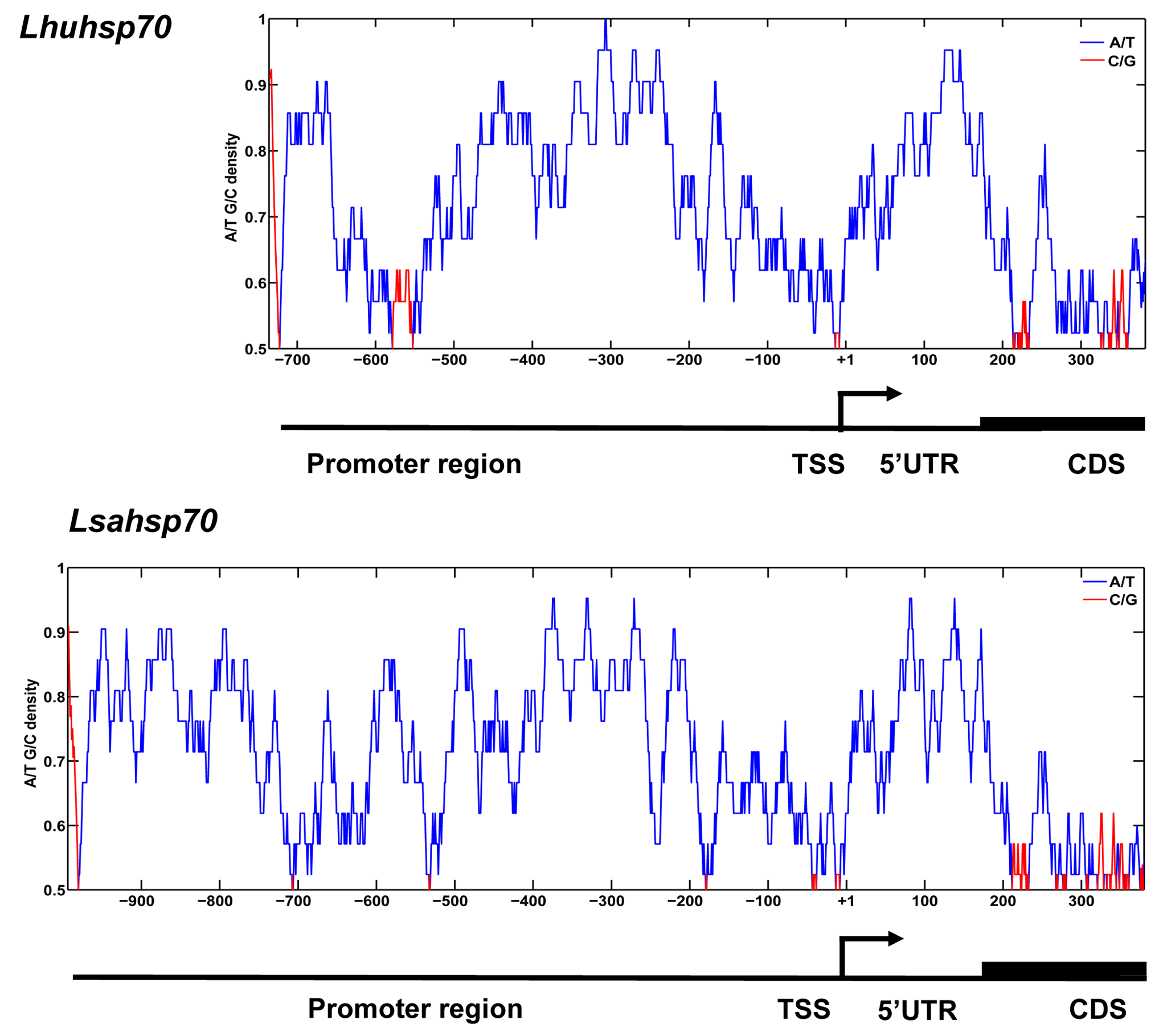

Supplement: Figure S2 — A+T content of the promoter regions of Lhuhsp70 and Lsahsp70 . Sliding-window size is 100 bp. “TSS”: the transcription start site; “5′UTR”: 5′ unstranscribed region; “CDS”: coding sequence. (TIF) [file pone.0020308.s002.tif]

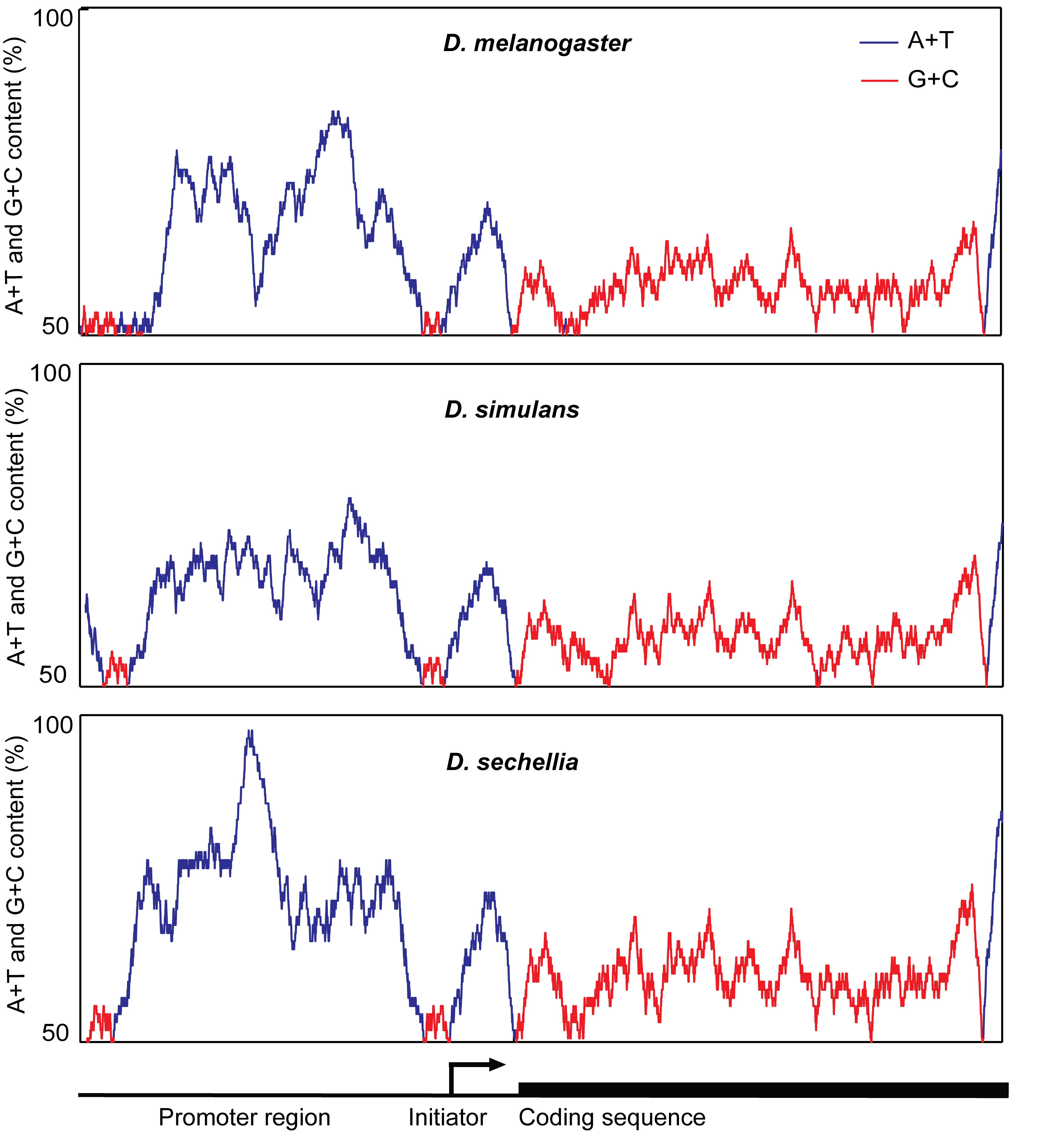

Supplement: Figure S3 — A+T content of the promoter and coding region of hsp70Ba of three Drosophila species. Sliding-window size is 40 bp. (TIF) [file pone.0020308.s003.tif]
