# Supplementary material for: Evolution of Hsp70 Gene Expression: A Role for Changes in AT-Richness within Promoters
Source: PLoS One. 2011 May 31;6(5):e20308. doi: 10.1371/journal.pone.0020308 (PMC3105046; doi:10.1371/journal.pone.0020308)
Supplement: Table S1 — Sequences of four HSEs in Lhuhsp70 and Lsahsp70. (DOC) [file pone.0020308.s004.doc]

Table S1. Sequences of four HSEs in *Lhuhsp70* and *Lsahsp70*

| HSEs | Sequences | Start position from transcription start site |
| --- | --- | --- |
| *LhuHsp70* gene | | |
| HSE1 | GTTCTCGAATATTCTCATGTGCTTG | -38 |
| HSE2 | TGACTTGAACATTCGAGAAATTTCC | -70 |
| HSE3 | GTTCGAGAAAATTCTCCTATTGTTG | -125 |
| HSE4 | TCTCTAGAAAATTCGAGGTAAATTT | -200 |
| *LsaHsp70* gene | | |
| HSE1 | TTTCGTGAACATTCCTATCAACTGG | -38 |
| HSE2 | ACACTCGAATATTCAAGAAAGTTCT | -70 |
| HSE3 | TGCCAAGAAAATTCACATGGGATTG | -620 |
| HSE4 | TAGTAAGAAAATTCCAGAATTCATC | -645 |
