# Supplementary material for: Evolution of Hsp70 Gene Expression: A Role for Changes in AT-Richness within Promoters
Source: PLoS One. 2011 May 31;6(5):e20308. doi: 10.1371/journal.pone.0020308 (PMC3105046; doi:10.1371/journal.pone.0020308)
Supplement: Table S2 — Primer sequences used in Materials and methods. (DOC) [file pone.0020308.s005.doc]

Table S2. Primer sequences used in *Materials and methods*

| Primer name | Primer sequences (5’ to 3’) |
| --- | --- |
| Genome- walking amplification of *Lhuhsp70* gene promoter region | |
| TSP1-Lhu | CCAGGTACCATTAGATGCGGTTGT |
| TSP2-Lhu | GCCCGCTAGCTTTGAAATAAATAAAC |
| TSP3-Lhu | CCAAGCTAGCGATAAAACTCTCACA |
| Genome- walking amplification of *Lsahsp70* gene promoter region | |
| TSP1-Lsa | CCAGGTACCATTAGATGCGGTTGT |
| TSP2-Lsa | GCCCGCTAGCTTTGAAATAAATAAAC |
| TSP3-Lsa | CCAAGCTAGCGATAAAACTCTCACA |
| Amplifying wild-type promoter region of *Lhuhsp70* gene and introduce restriction enzyme site for *BglII* and *KpnI* | |
| LhuHsp70F | CCAGGTACCATTAGATGCGGTTGT |
| LhuHsp70R | GAAGATCTGGAAGACACCAACGCAG |
| Amplifying wild-type promoter region of *Lsahsp70* gene and introduce restriction enzyme site for *BglII* and *KpnI* | |
| LsaHsp70F | CGGGGTACCGTAGAAATATCATGCTC |
| LsaHsp70R | GGGAGATCTGTTGAGTGTAGTTTGTAT |
| Amplifying upstream region of *ATRS1* with prime pair Lsahsp70F / ATRS1R, and downstream region with primer pair ATRS1F / LsaHsp70R to obtain deletion of *ATRS1*. *Pac1* restriction sites are underlined | |
| ATRS1R | GGGTTAATTAAGTTGAGTGTAGTTTGTAT |
| ATRS1F | CATTAATTAACTTGAGTGTTTCGTGAAC |
| Amplifying upstream region of *ATRS2* with prime pair Lsahsp70F / ATRS2R, and downstream region with primer pair HSE2-3F/ ATRS2F, to obtain deletion of *ATRS2*. *Pac1* restriction sites are underlined | |
| ATRS2R | GGGTTAATTAAGTTGAGTGTAGTTTGTAT |
| ATRS2F | CATTAATTAACTTGAGTGTTTCGTGAAC |
| Primers to amplify sequence fragment with 75% A+T from intergenic region of *D. melanogaster* genome to replace *ATRS1* | |
| ATRS1_75%F | CCTTAATTAATAAAGAAGAAGGTGGCAAAA |
| ATRS1_75%R | CCTTAATTAAAAAAAAAATTAAAAATATTA |
| Primers to amplify sequence fragment with 50% A/T from intergenic region of *D. melanogaster* genome to replace *ATRS1* | |
| ATRS1_50%F | CCTTAATTAATATATACAAATATCCATATA |
| ATRS1_50%R | CCTTAATTAACCCCAGTAAATTTTTAGCAA |
| Primers to amplify sequence fragment with 65% A/T from intergenic region of *D. melanogaster* genome to replace *ATRS2* | |
| ATRS2_65%F | CCTTAATTAACTTTCGAGAGCTTTCTCTCT |
| ATRS2_65%R | CCTTAATTAATTTAATTAATTGACAATATA |
| Primers to amplify sequence fragment with 50% A/T from intergenic region of *D. melanogaster* genome to replace *ATRS2* | |
| ATRS2_50%F | CCTTAATTAAATTGTTCCGCAGCACTTTCG |
| ATRS2_50%R | CCTTAATTAATGTCCTGCCTTTATATCTCG |
| Primers for site-directed mutagenesis of Zeste binding sites via overlapping extension PCR | |
| zestemotif1F | GCTGAAGTTTTTAGATGAATTAAAG |
| zestemotif1R | TCTAAAAACTTCAGCTCCTTCAAAC |
| zestemotif2F | TCAGTAGTTCACCATTAGATGTTTG |
| zestemotif2R | TCTAATGGTGAACTACTGAGTGCAT |

Underlined sequences are restriction sites for enzymes.
